# Use of Meropenem in a Tertiary Pediatric Hospital in Costa Rica and Its Role in the Era of Antimicrobial Stewardship

**DOI:** 10.7759/cureus.15809

**Published:** 2021-06-21

**Authors:** Constanza Chacón-González, Daniel Rivera-Salgado, Helena Brenes-Chacón, Gabriela Naranjo-Zuñiga, María L Ávila-Aguero

**Affiliations:** 1 Pediatrics, Hospital Nacional de Niños "Dr. Carlos Sáenz Herrera", San José, CRI; 2 Medicine, Universidad de Ciencias Médicas (UCIMED), San José, CRI; 3 Medicine, Universidad de Costa Rica, San José, CRI; 4 Pediatric Infectious Diseases, Hospital Nacional de Niños "Dr. Carlos Sáenz Herrera", San José, CRI; 5 Pediatric Infectious Diseases, Hospital Nacional De Niños "Dr. Carlos Sáenz Herrera", San José, CRI; 6 Pediatric Infectious Diseases, Center for Infectious Disease Modeling and Analysis, Yale School of Public Health, New Haven, USA

**Keywords:** antimicrobial stewardship, antibiotic resistance, meropenem, pediatrics, therapeutics

## Abstract

Background

Understanding antibiotic profiles and their resistance patterns can improve hospital quality care and optimize clinical outcomes. This paper characterizes the use of meropenem in the National Children’s Hospital of Caja Costarricense del Seguro Social (CCSS) in Costa Rica, and its role in antibiotic stewardship.

Methods

This is a retrospective observational study from hospitalized patients under 13 years of age that received meropenem as part of their treatment. Patients were identified through medical and pharmacy records. Data was summarized using frequencies and percentages for categorical variables, means and standard deviations for normally distributed continuous variables, and medians with interquartile ranges (IQR) for non-normally distributed continuous variables.

Results

A total of 181 of the 309 selected patients met inclusion criteria. Median age was 21 months (IQR: 4.0-79.0). Mean length of stay was 31 days (16.0-58.0). The most frequent diagnosis was septic shock (29%). 87% of patients received at least one antibiotic prior to receiving meropenem; 71% of patients received a second antibiotic simultaneously with meropenem. In 113 (62%) cases, meropenem was prescribed as empirical therapy. The most frequent isolate was extended-spectrum ß-lactamase *Escherichia coli *(24%). 74% of patients who received meropenem as targeted therapy had a favorable outcome.

Conclusions

Meropenem can be used as monotherapy for complicated, multi-drug resistant, gram negative, bacterial infections, due to its susceptibility profile, convenient dosing schedule, and minimum adverse effects. However, it should be restricted to cases where no other drug is available in order to safeguard its value.

## Introduction

Antibiotic resistance has become one of the main problems in healthcare settings worldwide. As resistance patterns continue to emerge rapidly and only few new antimicrobial drug classes have been developed and approved, there is a need to implement practices to avoid antibiotic overuse [1, 2]. Cumulative susceptibility data alone is no longer reliable to guide antibiotic choice [3]. As a result, implementation of antimicrobial stewardship programs has become more common among hospitals around the globe.

Antimicrobial stewardship is a strategy endorsed by the World Health Organization (WHO) and the Infectious Disease Society of America (IDSA) among others, to improve hospital quality care and optimize clinical outcomes while simultaneously minimizing adverse clinical events, new resistance patterns, and opportunistic infections [1, 3-7].

Meropenem is an antibiotic of the carbapenem family, a beta-lactam that acts inhibiting cell wall synthesis by binding to penicillin-binding proteins. It has a time-dependent, broad spectrum coverage, allowing it to act against gram-positive and gram-negative organisms, as well as anaerobes. Its structural stability makes it resistant to extended-spectrum and AmpC ß-lactamases [8-11]. It is recommended for treatment of infections caused by bacteria resistant to standard therapy, in cases such as severe intra-abdominal sepsis, pneumonia, bone and joint infections, bacteremia with beta-lactamase producing bacteria, and septic shock [1, 3, 8, 10].

Few reports of the clinical use of meropenem in children have been published and therefore it is important to document this clinical experience [8-10, 12-14].

The aim of this paper is to characterize the use of meropenem in the pediatric healthcare setting in the only tertiary National Children’s Hospital in Costa Rica, and its role during the era of antimicrobial stewardship.

## Materials and methods

This is a retrospective observational study conducted at the National Children’s Hospital “Dr. Carlos Sáenz Herrera”, the tertiary teaching hospital for pediatrics in Costa Rica and part of the social security network “Caja Costarricense del Seguro Social” (CCSS). Hospitalized patients under 13 years of age that received meropenem as part of their treatment between June 30th 2010 and January 31st 2016 were included.

To include patients, medical and pharmacy records were consulted. Chart review allowed to include age, sex, length of hospital stay (LOS), ward of admission, diagnosis, reason for antibiotic prescription (whether empirical or targeted), length of treatment (LOT), adverse effects, culture results, complications, and infectious disease division consult before prescription.

A total of 1009 patients received meropenem in the established time period. A sample of 309 patients was obtained using Raosoft®’s sample size calculator (Raosoft, Inc., Seattle, WA), aiming for a 95% confidence interval (CI) and an alpha error of 5%. A total of 181 patients met inclusion criteria of being under 13 years of age receiving meropenem as part of their treatment in our hospital, therefore an 86% CI and 6.6% alpha error was obtained. Microsoft® Excel (for Mac 2017, v. 15.40) (Microsoft® Corp., Redmond, WA) was used for data entry and analysis. GraphPad (Prism 8 for Mac OS, v. 8.4.3) (GraphPad Software, San Diego, CA) was used for statistical analysis. Data were summarized using frequencies and percentages for categorical variables, means and SDs for normally distributed continuous variables, and medians with interquartile ranges for non-normally distributed continuous variables.

Ethics committee approval to conduct the study was obtained from the Local Ethics and Research Committee of the National Children’s Hospital “Dr. Carlos Sáenz Herrera”, under the CEC-HNN-028-2017 approval project.

Patients or the public were not involved in the design, recruitment, conduct, reporting, or dissemination plans of our research.

## Results

**Figure 1:**
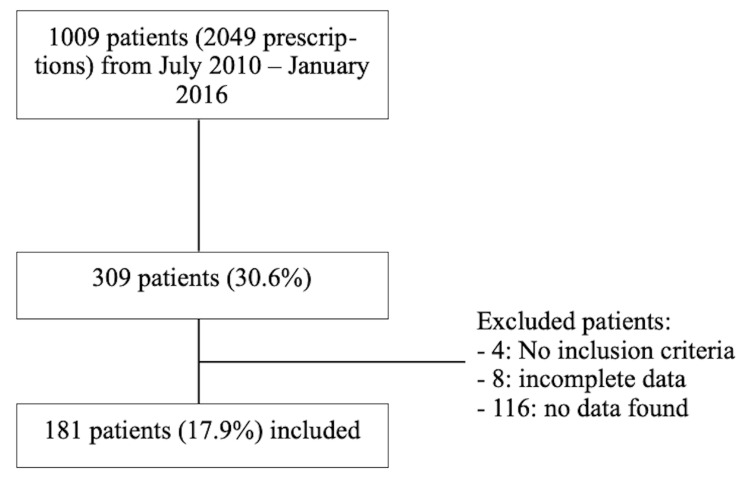
Study subject selection

The hospital receives an average of 14,000 hospitalizations per year. Of the 1009 hospitalized patients who received meropenem as part of their treatment between June 2010 and January 2016, 309 were randomly selected as sample of patients, with 181 meeting inclusion criteria (Figure 1). Of those included, median age was 21 months (IQR 25-75, 4.0-79.0), with 98 (54%) males (Table 1).

**Table 1:** Clinical characteristics of patients hospitalized receiving meropenem Clinical characteristics of patients included in the study. Categorical data are expressed as frequencies (%) and continuous data are expressed as median [25%-75% interquartile range]. LOS, Length of Stay; ID, Infectious Diseases Division; LOT, Length of Treatment.

	Patients (n = 181)
Age (months)	21.0 (4.0-79.0)
Sex (male)	98 (54.1)
LOS, days	31.0 (16.0-58.0)
Patients receiving previous antibiotics	157 (86.7)
ID consult before starting meropenem	83 (45.9)
Empiric prescription	113 (62.4)
Targeted prescription	68 (37.6)
Discontinuation of meropenem with negative cultures	15 (8.3)
LOT, days	10.0 (6.0-15.5)
Mortality	50 (27.6)

**Figure 2:**
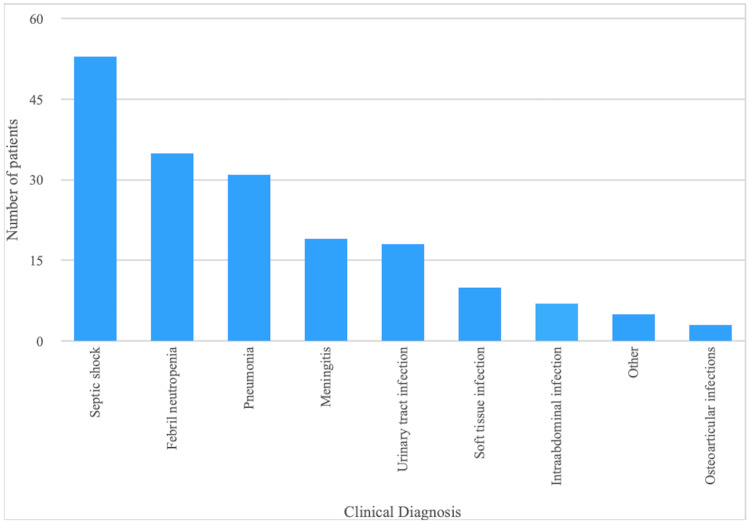
Clinical diagnosis to start meropenem Clinical diagnosis at the moment of meropenem prescription. The X axis represents the different clinical diagnosis, and the Y axis the number of patients. Numbers above columns represent the frequency (%) for each diagnosis.

Median length of hospitalization stay was 31.0 days (16.0-58.0), with 61 (33.7%) admitted to the pediatric intensive care unit (PICU), and 120 (66.3%) at ward, mainly distributed in hematology-oncology (21%), infectious diseases (13%), and neonatology (10%). The most frequent diagnosis for which meropenem was prescribed was septic shock (29%), followed by febrile neutropenia (19%), and pneumonia (17%) (Figure 2). In 10% of these cases meropenem was prescribed on the first day of admission, while 40% received it during the first seven days of hospitalization, and 59% of cases on day 14.

According to pharmacy records, 87% of patients had received at least one antibiotic prior to meropenem, including amikacin (61%), vancomycin (52%), cefotaxime (43%), and ceftazidime (43%). 71% of patients received a second antibiotic simultaneously with meropenem; most frequently vancomycin (79%) and amikacin (40%).

In 46% of cases a consult to the infectious diseases team was made. In 113 (62%) of cases meropenem was prescribed as empirical therapy, having cultured blood and tissue samples prior to initiation antibiotics. Despite 73% of these cultures were negative, meropenem was discontinued only in 15 (8.3%) cases.

The most frequent isolate for which meropenem was used as targeted therapy was extended-spectrum ß-lactamase *Escherichia coli* (24%), followed by *Pseudomonas aeruginosa* (17%), and *Enterobacter spp.* (15%).

There were no adverse events that required discontinuation of meropenem. 74% of patients who received meropenem as targeted therapy had a favorable outcome, measured as no death, no relapse, and negative cultures. During the study period, mortality was documented in 50 (24%) of patients. Although causes of death were not specified, none of them were directly related to meropenem use.

## Discussion

We conducted a retrospective single center study monitoring the use of meropenem in hospitalized settings. We found that in our hospital meropenem was used in patients with underlying disease or admitted to the PICU, but in the most part as part of empirical therapy regimens.

In the study median age of patients was less than 24 months of age, and most of these patients had a long length of hospitalization, with one-third of them requiring PICU admission.

Meropenem is an effective antimicrobial used in the setting of severe infection: febrile neutropenia, pneumonia, intraabdominal infections, meningitis, urinary tract infections, and early or late onset sepsis in neonates [3, 12, 13]. In our study, the most common diagnoses were septic shock, febrile neutropenia, and pneumonia, consistent with that reported in the literature. At that time, the hospital did not have clinical guidelines for management of fever and neutropenia in hematology-oncology patients, which were implemented by 2018, thus the use of broad-spectrum antibiotics such as meropenem was not regulated.

In our study 62% of patients received meropenem as part of an empirical therapy regimen, and most patients received other antibiotics prior to meropenem, such as amikacin, cefotaxime, or ceftazidime. Meropenem allow coverage for both Gram negative and positive bacteria, making it an appealing choice for empirical therapy [11]. However, data support that it should be used judiciously and reserved only for cases where ß-lactamase production is suspected or confirmed, particularly in cases of *Klebsiella* *spp.* or *Enterobacter* *spp*. [3, 12]. In this study, the most frequent isolate was *E. coli* producing extended spectrum ß-lactamase, followed by *P. aeruginosa* and *Enterobacter* *spp*. However, only 38% of prescriptions were targeted to a particular microorganism, and less than 10% patients had an antibiotic change once negative cultures were reported.

One of the advantages of meropenem is that its broad-spectrum characteristics allow it to be used as monotherapy in most settings [12, 14, 15], unlike what was evidenced in our study, where only 29% of patients received meropenem alone. Berkowitz et al. identified no difference in clearance of bacteremia or mortality comparing patients who received empiric monotherapy and combination antimicrobial therapy with aminoglycosides when managing Enterobacteriaceae bacteremia [16]. However, exceptionally, it can be combined with vancomycin as second-line therapy for multi-resistant early or late onset sepsis in newborns, or in other scenarios such as septic shock, as seen in 71% of our patients [12]. A high incidence of gram-positive organisms such as Staphylococcus sp. specially among patients with central venous catheters or other interventions in the PICU, could explain the decision to not start meropenem as monotherapy in some instances.

Meropenem is a well-tolerated medication that rarely requires discontinuation due to adverse effects, as was shown in our study [9, 11, 13, 17].

Although meropenem has important advantages, it is not risk free. Its constant use has allowed an increase in multi-resistant gram-negative bacterial infections and colonization, particularly with *P. aeruginosa* [15, 18]. Recent data of our hospital antibiogram, exhibit good susceptibility not only to meropenem but also to third generation cephalosporins in the treatment of infections caused by Enterobacteriaceae, which does not justify the use of meropenem as the first option for empiric therapy [19]. Additionally, meropenem has been associated with changes in the gut microbiota of pre-term infants due to its use in NICU settings [20, 21]. This has led to a bacterial dysbiosis, increasing the risk of developing necrotizing enterocolitis and other infections, diabetes, allergies, obesity, and inflammatory bowel disease among others [21, 22]. The overuse and misuse of meropenem has also shown an increased carriage of antibiotic-resistant genes in some bacteria, increasing resistance to other antibiotic classes such as fluoroquinolones, macrolides, and other ß-lactams [20]. This was not analyzed in our study, but should be taken into consideration for future research.

Resistance rates for meropenem are low, but they still pose an important threat, particularly associated with high mortality rates [18, 23]. According to a multicenter survey done in the United States of America (USA), resistance rates range from 1.1 to 2.5%, while in Taiwan rates vary from 7-12% [3]. Carbapenem-resistant gram-negative bacteria were identified in 1.7% of discharged patients from NICU settings [15]. In our study, of the 73% of cases with negative results, only 8.3% discontinued meropenem. A major problem at that time, was that our hospital did not have an Antimicrobial Stewardship Program (ASP), thus any physician could prescribe meropenem without restriction and there was no prospective audit or feedback. Starting 2019, a Costa Rican ASP was implemented, regulating the prescription of broad-spectrum antibiotics such as meropenem.

Antimicrobial stewardship programs are an effective tool to guide physicians in decision-making regarding antibiotics [2, 7]. Frequent reevaluation of empiric choices and de-escalating or discontinuing treatment once laboratory and antibiotic susceptibility results are available, while correlating with local guidelines, is an effective way to prevent multi-drug resistance [2, 4-7, 12]. Gonzales et al. conducted a study where a triple ß-lactam combination was used for MRSA N315 strains in vitro, suggesting it could be an effective alternative to emerging resistance meanwhile using existing antibiotics. They claim that by using inhibitors of cell wall synthesis there is a synergistic activity that can have clinically significant outcomes, as well as reducing toxic effects caused by high meropenem concentrations [24]. However, more evidence is needed.

As antibiotic resistance worsens, fewer options are available in terms of antimicrobial therapy. The correct use of meropenem will allow us to keep an open door to treat severe infections associated with multi-drug resistance and high mortality rates. Interdisciplinary teams with clinical pharmacists and infectious diseases (ID) specialists are an important tool in antimicrobial stewardship programs [2, 4, 5, 7]. It is necessary that the multidisciplinary approach continues to guide treatment most effectively while educating physicians in making better therapeutic decisions. In our study, only one-third of cases consulted ID prior to initiating meropenem.

The main limitation to this study was that the sample was obtained from a single hospital, leading to different biases. Nonetheless, this is the first study that analyzes the use of meropenem in pediatric or adult populations in Costa Rica. Despite the small number of patients receiving meropenem in comparison to the annual hospitalizations in our hospital, this analysis allowed us to develop new antibiotic stewardship programs and modify established protocols. Also, there was no general consensus for septic shock, therefore errors in identification of shock could have played a role in antibiotic misuse. In addition, cause of death was not specified when considering mortality, making it difficult to differ whether it was due to treatment failure, multi-resistance patterns, or any other cause. Finally, this study did not analyze meropenem dosage as a variable, so despite most patients having a favorable outcome, it is an area that requires further investigation in order to optimize treatment.

Additionally, limitations still exist concerning the use of meropenem. Determining whether pathogens are carbapenemase-producers is an essential yet expensive advancement. When these bacteria are identified, reserved antibiotics should be used, limiting their prescription to these specific cases and preventing medication misuse [23].

Further research is needed in developing countries such as Costa Rica. The recent creation of antimicrobial stewardship programs is a stepping stone in the process to influence a better use of antibiotics. More data is needed to address the impact of the program in our facility.

## Conclusions

Meropenem is a broad spectrum antibiotic, making it an excellent choice for use in patients with suspected or confirmed severe infections. However, its use should be restricted to cases where no other drug is available for the shorter possible time to safeguard its value. In our setting, we documented that meropenem was mainly used in patients with underling diseases, especially in those with an immunocompromised status, and patients acutely ill in the PICU. Nevertheless, improvement in monitoring and discontinuation of therapy in some cases is still necessary.

Local and nationwide investment in antibiotic education and guided decision-making is crucial in order to give physicians the tools to steer their choices away from multi-drug resistance patterns. Establishing interdisciplinary antibiotic stewardship programs has proven to be effective in hospital-care settings and should be encouraged in order to achieve improved patient safety. The emergence of Antimicrobial Stewardship Programs in Latin America is a high value tool in combating antimicrobial resistance.
